# Clinical Efficacy and Safety of Trans-Sacral Epiduroscopic Laser Decompression Compared to Percutaneous Epidural Neuroplasty

**DOI:** 10.1155/2019/2893460

**Published:** 2019-01-14

**Authors:** Bong Ju Moon, Seong Yi, Yoon Ha, Keung Nyun Kim, Do Heum Yoon, Dong Ah Shin

**Affiliations:** ^1^Department of Neurosurgery, Chonnam National University Hospital and Medical School, Gwangju 61469, Republic of Korea; ^2^Department of Neurosurgery, Yonsei University College of Medicine, Severance Hospital, Seoul 03722, Republic of Korea

## Abstract

Percutaneous epidural neuroplasty (PEN) is an effective and safe procedure for herniated lumbar disc (HLD). Although PEN has an advantage of adhesiolysis, this procedure cannot decompress the protruded disc. Recently, trans-sacral epiduroscopic laser decompression (SELD) for HLD has been introduced as a promising alternative methodology. This study evaluated the clinical efficacy and safety of SELD compared to PEN, as well as the change in protruded disc volume after SELD through pre- and postoperative magnetic resonance imaging (MRI), in patients with HLD. Thirty consecutive patients underwent SELD (SELD group), and 45 patients underwent PEN (PEN group). The Visual Analog Scale (VAS) for leg pain; Oswestry Disability Index (ODI); 12-Item Short-Form Health Survey (SF-12); preoperative and postoperative 4-, 12-, and 24-week Macnab criteria; and preoperative and 24-week postoperative lumbar spinal MRIs after SELD were obtained. There was no significant difference in age, sex, duration of symptoms, and the distributions of disc level between the two groups (all *P* > 0.05). Between the SELD and PEN groups, preoperative VAS, ODI, and SF-12 scores had no significant differences. However, the VAS, ODI, and SF-12 scores improved significantly after the procedures by postoperative week 24 in each group (all *P* < 0.05). Furthermore, improvements of VAS, ODI, SF-12, and success rate of Macnab criteria in the SELD group were better than those in the PEN group (all *P* < 0.05). The protruded disc volume after SELD decreased significantly (*P*=0.034). All clinical and functional outcomes of patients undergoing SELD and PEN for HLD improved following the procedures. Notably, SELD was superior to PEN regarding the degree of improvement in clinical and functional outcomes. Therefore, we suggest that SELD can be used as an effective alternative to PEN to provide improved clinical and functional outcomes in patients with HLD.

## 1. Introduction

Percutaneous epidural neuroplasty (PEN) is a minimally invasive therapy for spinal diseases [1–4]. Although it was originally developed to treat postoperative adhesion, PEN has also been used for targeted drug delivery, epidural scarring, and neural decompression [1, 4]. Additionally, the procedure has shown good clinical outcomes as a herniated lumbar disc (HLD) treatment when compared to physiotherapy [3]. To alleviate radicular or lower back pain in patients with HLD, PEN can ameliorate aberrant adhesion and deliver drugs to areas with pathology, including the nerve root and disc [4, 5]. However, a disadvantage of the PEN procedure is that it cannot decompress the herniated disc. Percutaneous lumbar laser ablation is an alternative solution that addresses this problem [6].

The first clinical percutaneous lumbar laser disc decompression was reported in Europe by Choy and colleagues in 1986 [7]. In percutaneous lumbar laser ablation, laser energy is delivered to the herniated lumbar disc through a thin optical fiber. The absorption of applied laser energy vaporizes the water content of the nucleus pulposus and then changes its protein structure [8, 9]. The subsequent reduction in the nucleus volume results in reduced intradiscal pressure, which in turn leads to the decompression of entrapped nerve roots [6].

Epiduroscopy is also a new minimally invasive technique for the management of spine-originating diseases. The epiduroscopic view is beneficial for the management of lumbar spine disease because it provides direct visualization of spinal pathology and can be used simultaneously with PEN. Similarly, laser decompression with epiduroscopy can be a more effective method for treating intraspinal pathologies, such as a herniated nucleus pulposus and painful microscopic adhesions [6].

Trans-sacral epiduroscopic laser decompression (SELD) is a new technique that has been reported to be effective in treating lumbar disc herniation [10]. However, the few studies that have investigated this procedure involving a sacral hiatus approach have only reported on clinical outcomes [10–13]. Therefore, the goal of this study is to evaluate the clinical efficacy and safety of SELD compared to PEN, as well as the change in disc volume after SELD through pre- and postoperative magnetic resonance imaging (MRI).

## 2. Materials and Methods

### 2.1. Study Design and Patients

Approval for the current study was granted by the Institutional Review Board of our institute (approval number, 1-2014-0049). A total of 75 patients who signed an informed consent form were included in this study between March 2013 and December 2014. All patients had leg pain that was refractory to conservative management, such as medication and physiotherapy, for more than 6 weeks. Each patient also had a single level HLD that was checked by MRI. Exclusion criteria were previous spinal invasive procedure or operation, instability, spondylolisthesis, ossification of the posterior longitudinal ligament, and other traumatic injuries, as well as underlying systemic diseases like rheumatoid arthritis and systemic lupus erythematosus.

**Figure 1:**
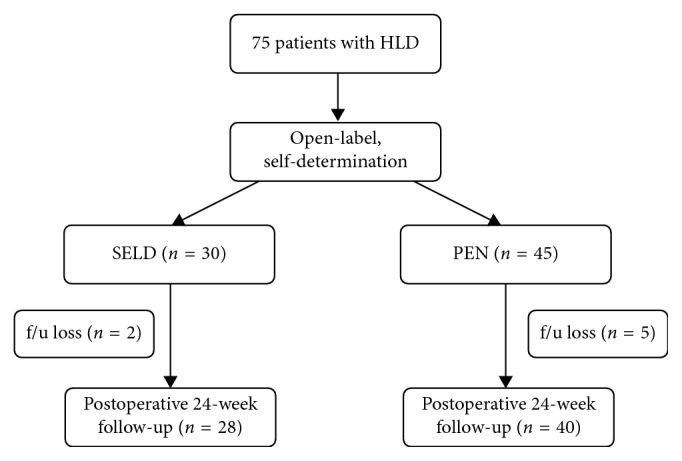
Flow chart showing the number of patients undergoing treatment by SELD and PEN in the current study. HLD = herniated lumbar disc; SELD = trans-sacral epiduroscopic laser decompression; PEN = percutaneous epidural neuroplasty; f/u = follow-up; POD = postoperative duration.

The choice of surgical procedure was made by the patients in this open-label study. Among the 75 patients with HLD, 30 patients decided to undergo SELD, and the other 45 patients decided to undergo PEN for the treatment of HLD. However, 2 of the 30 patients in the SELD group and 5 of the 45 patients in the PEN group were not included in the last follow-up period (Figure 1).

### 2.2. Surgical Procedures

#### 2.2.1. Percutaneous Epidural Neuroplasty (PEN)

PEN was performed under fluoroscopy as a one-day treatment in a sterile operating room, and blood pressure, pulse rate, and pulse oximetry were monitored. After the patient was in the appropriate prone position, the needle insertion area around the sacral hiatus was injected with 1% lidocaine (Yooyoung, Seoul, Korea). An RK epidural needle (Epimed International, Johnstown, NY, USA) was introduced into the caudal epidural space under fluoroscopic guidance. When the position of the needle was confirmed to be in the epidural space, a lumbar epidurogram was performed using approximately 5 mL of iodinated contrast medium (IOBRIX, ACCUZEN, Seoul, Korea). Confirmation of filling defects was achieved by observing the contrast agent flow. We also confirmed that there was no intravenous or subarachnoid space penetration by the needles. If the needle position was found to be incorrect, the position of the needle was altered to address the problem. After adequate confirmation with the epidurogram, a Racz catheter (Epimed International, Johnstown, NY, USA) was advanced through the RK needle to the filling defect area or pathological site as determined by MRI. Once the needle was in the final location in the lateral or ventral epidural space, adhesiolysis was performed. After adhesiolysis, a minimum of 3 mL of contrast medium was injected. If there was no subarachnoid, intravascular, or another extra epidural filling, and satisfactory filling was obtained in the epidural and targeted regions, 6 mL of 0.2% preservative-free ropivacaine (AstraZeneca, Seoul, Korea) containing 1,500 units of hyaluronidase (Huons, Seoul, Korea) and 2 mL of dexamethasone (Huons, Seoul, Korea) were injected [2].

#### 2.2.2. Trans-Sacral Epiduroscopic Laser Decompression (SELD)

**Figure 2:**
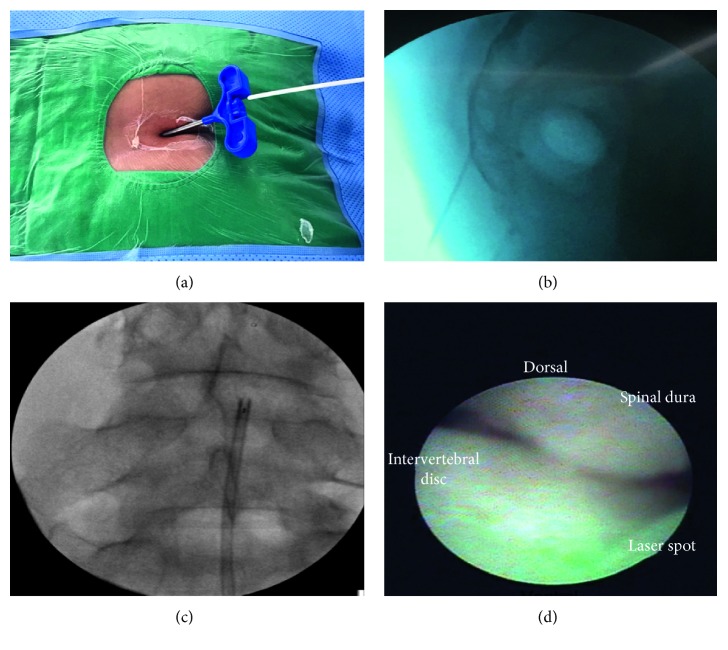
SELD procedure. (a) Insertion of SELD catheter through a needle introduced into the sacral hiatus; (b) fluoroscopic epidurogram at caudal epidural space; (c) placing SELD catheter at disc level L4/5; (d) epiduroscopic view of epidural space. SELD = trans-sacral epiduroscopic laser decompression; Dorsal = dorsal side of the patient (ventral side of thecal sac); POD = postoperative duration.

The SELD procedure (Figure 2) was performed with the patient in a prone position, and physiological parameters were monitored, similar to the PEN procedure. At the area of the sacral hiatus, 1% lidocaine (Yooyoung, Seoul, Korea) was administered, and a 1 cm longitudinal incision was made in the sacral hiatus region. An epidural needle was used to puncture the sacral hiatus, and a steerable catheter (Biovision 3.0 epidural catheter; Biovision Technologies, Golden, CO, USA) was inserted into the epidural space through the introducing needle. A flexible epiduroscope (NeedleView CH, Lutronics, Goyang-si, Gyeonggi-do, Korea) was steered using Biovision 3.0 epidural catheter to the location of the lesion as determined by MRI. Epidural saline solution was used to irrigate and clear the area visualized on the endoscopic video screen as well as to cool the ablation site. Through the epiduroscope, we could see the herniated disc in addition to adhesive bands, inflammatory tissues, fibrous connective tissues, and adipose tissue around the dura and nerve root. Through the epiduroscope (NeedleView CH, Lutronics, Goyang-si, Gyeonggi-do, Korea), we could see that the disc was reduced by the Nd : YAG (neodymium-doped yttrium aluminum garnet) laser system (ACCUPLASTI, Lutronic, Goyang-si, Gyeonggi-do, Korea). The laser system output power range was from 2.5 W (0.5 J, 5 Hz) to less than 5 W (0.6 J, 8 Hz or 0.5 J, 10 Hz). After laser ablation, 6 mL of 0.2% preservative-free ropivacaine (AstraZeneca, Seoul, Korea) containing 1,500 units of hyaluronidase (Huons, Seoul, Korea) and 2 mL of dexamethasone (Huons, Seoul, Korea) were injected, as was done for the PEN procedure.

### 2.3. Data Collection and Statistical Analysis

**Figure 3:**
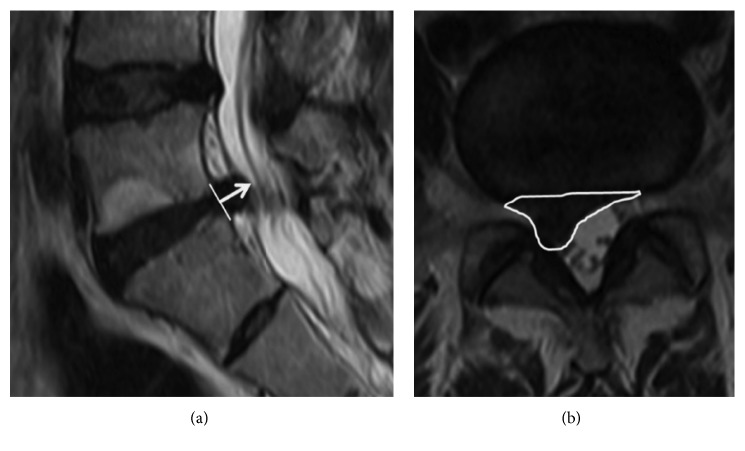
Protruded disc volume. The disc volume was calculated by multiplication of the height and the area. (a) The height (white arrow) in T2 sagittal MRI; (b) the area (white ellipse).

The Visual Analog Scale (VAS) for leg pain, Oswestry Disability Index (ODI), 12-Item Short-Form Health Survey (SF-12), and preoperative and postoperative 4-, 12-, and 24-week Macnab criteria were obtained for all patients. In addition, preoperative and 24-week postoperative lumbar spine MRIs were evaluated to identify changes in disc volume after SELD. Disc volume was calculated by the following equation: height (mm) × area (mm^2^). The height was defined as the maximal diameter of the disc space (Figure 3(a)). The area of disc protrusion was obtained by using a region of interest (ROI) defined with Centricity PACS software (GE Healthcare, Little Chalfont, BKM, England), as in Figure 3(b).

VAS, ODI, and SF-12 scores were analyzed by the repeated measures analysis of variance (RM-ANOVA) with Bonferroni correction (*P* < 0.0125). Success rates of Macnab criteria were analyzed by the chi-square test. The change in protruded disc volume between pre- and postoperative SELD measurements was analyzed by the Wilcoxon signed-rank test. All statistical analyses were performed using SPSS software version 18 (SPSS Inc., Chicago, IL, USA), and statistical significance was defined as *P* < 0.05.

## 3. Results

### 3.1. Demographics

There was no significant difference in age, sex, duration of symptoms, and the distributions of disc level between the two groups (all *P* > 0.05; Table 1).

### 3.2. Clinical Outcomes

**Figure 4:**
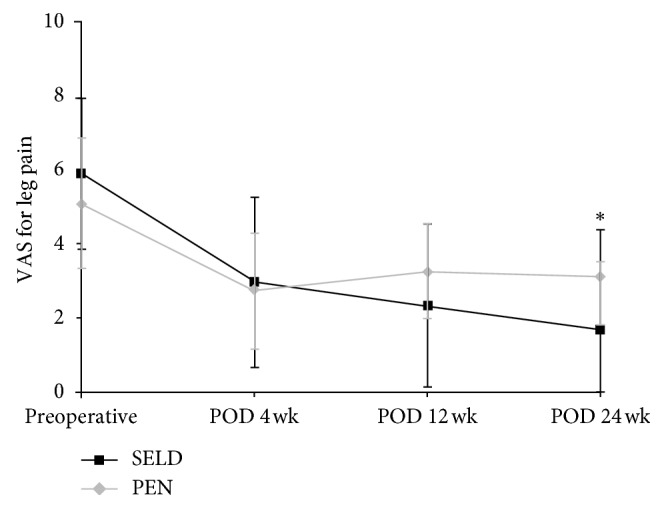
Comparison of VAS scores for leg pain between the SELD and PEN groups preoperatively and at 4, 12, and 24 weeks after treatment; ^*∗*^statistically significant differences were observed at 24 (*P*=0.001) weeks postoperatively. SELD = trans-sacral epiduroscopic laser decompression; PEN = percutaneous epidural neuroplasty; VAS = visual analog scale; POD = postoperative duration.

**Figure 5 fig5:**
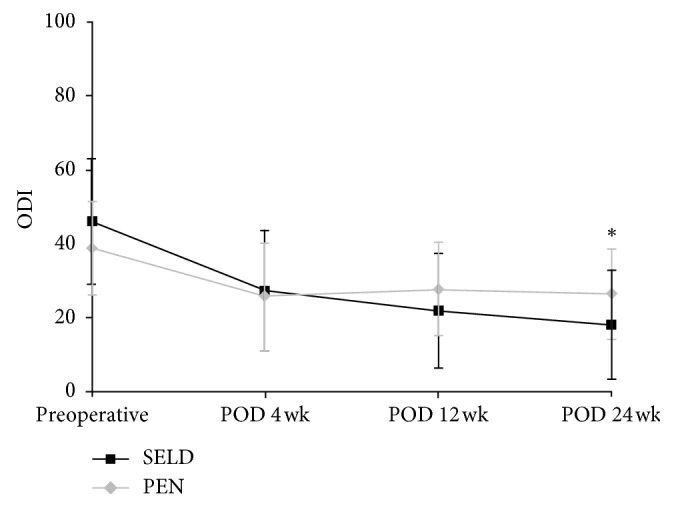
Comparison of ODI scores for leg pain between the SELD and PEN groups at preoperative weeks 4, 12, and 24; ^*∗*^statistically significant differences were observed at 24 weeks postoperatively (*P*=0.011). ODI = Oswestry Disability Index; SELD = trans-sacral epiduroscopic laser decompression; PEN = percutaneous epidural neuroplasty; POD = postoperative duration.

**Figure 6:**
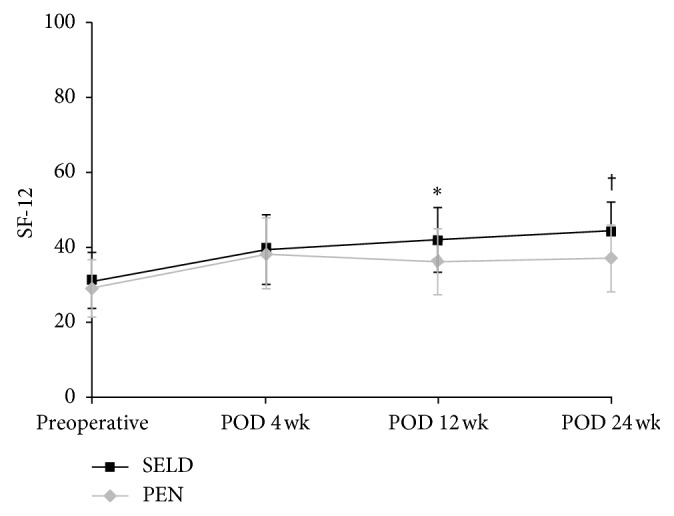
Comparison of SF-12 scores for leg pain between the SELD and PEN groups preoperatively and at 4, 12, and 24 weeks after treatment; ^*∗*^, ^†^statistically significant differences were observed at 12 (*P*=0.008) and 24 (*P*=0.001) weeks postoperatively. ODI = Oswestry Disability Index; SELD = trans-sacral epiduroscopic laser decompression; PEN = percutaneous epidural neuroplasty.

The results of the repeated measures analysis of variance (RM-ANOVA) for the changes in the VAS and ODI and SF-12 scores of the SELD and PEN groups at the four measurement time points are shown in Figures 4 [Fig fig5]–6. Between the SELD and PEN groups, the respective preoperative VAS (5.90 ± 2.02 vs 5.07 ± 1.77, *P*=0.079), ODI (46.07 ± 17.27 vs 38.9 ± 12.81, *P*=0.068), and SF-12 (31.08 ± 7.54 vs 29.05 ± 7.61, *P*=0.28) scores showed no significant differences (Figures 4 [Fig fig5]–6). However, within each group, the VAS and ODI scores showed a significant decrease, and the SF-12 score showed a significant increase, at postoperative week 24 (all *P* < 0.05) for both procedures. Notably, the SELD group showed better VAS improvement at postoperative week 24 than the PEN group (1.68 ± 1.79 vs 3.12 ± 1.26; *P*=0.001; Figure 4). The SELD group also showed better ODI improvement at postoperative week 24 than the PEN group (17.89 ± 14.82 vs 26.5 ± 12.1; *P*=0.011; Figure 5). Furthermore, the SF-12 scores of the SELD group were higher than those of the PEN group at postoperative week 12 (42.07 ± 8.62 vs 36.27 ± 8.58; *P*=0.008) and week 24 (44.30 ± 7.51 vs 37.22 ± 9.06; *P*=0.001) (Figure 6). Finally, the SELD group showed a significantly better success rate as measured by the Macnab criteria (success defined as “excellent” or “good” in Macnab criteria) at postoperative weeks 12 and 24 (*P* ≤ 0.001,  *P* ≤ 0.001, Table 2). There were no procedure-related complications such as nerve damage, dural puncture, increased intracranial pressure, or postoperative infection in either group.

### 3.3. Disc Volume Changes and Laser Output

**Figure 7:**
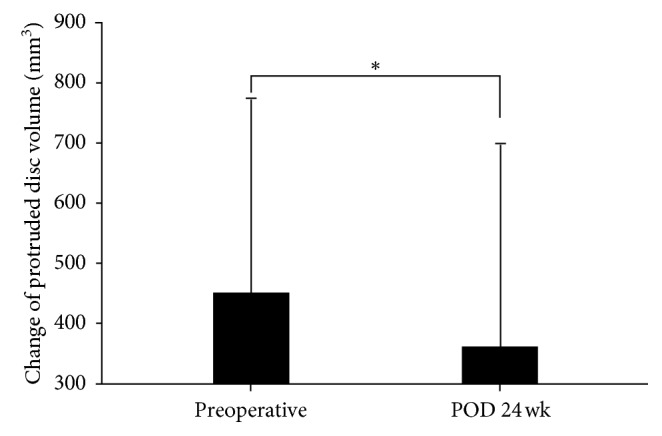
Change of protruded disc volume from preoperatively to after the SELD procedure. ^*∗*^Statistically significant differences were observed at 24 weeks postoperatively (*P*=0.034). SELD = trans-sacral epiduroscopic laser decompression; Preop = preoperative day; POD = postoperative duration.

**Figure 8:**
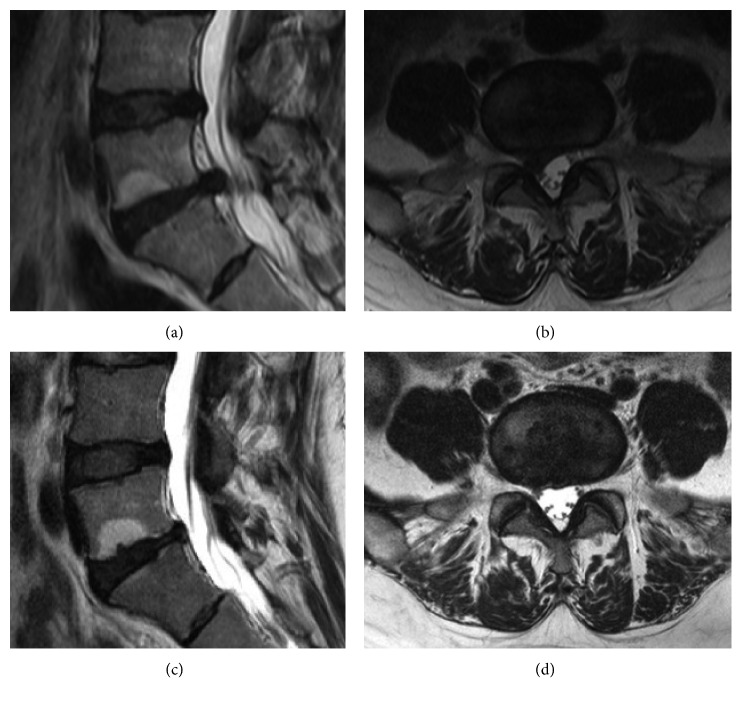
Case presentation regarding change of protruded disc volume after the SELD procedure. The 38-year-old man suffered from right leg pain with the protruded disc at L5/S1 level. After the SELD procedure, the volume of the protruded disc at the L5/S1 level decreased. (a) Preoperative sagittal T2 MR image; (b) preoperative axial T2 MR image; (c) postoperative sagittal T2 MR image; (d) postoperative axial T2 image. SELD = trans-sacral epiduroscopic laser decompression; MR = magnetic resonance.

In the patients who underwent the SELD procedure, the average protruded disc volume was 452.54 ± 322.08 mm³ preoperatively and 361.69 ± 339.10 mm³ at 24 weeks postoperatively, which translated to a significant decrease in the volume of 90 ± 244.25 mm³ (90 mm³ = 0.09 cc; *P*=0.034, Figures 7 and 8). The total laser output energy during the procedure was 776.25 ± 609.14 W.

## 4. Discussion

Previous studies have shown that PEN with or without epiduroscopy demonstrated superior effectiveness compared with both physical therapy and caudal epidural injections [3, 4]. Its advantages extend beyond adhesiolysis to include drug delivery to areas of pathology that occur in diseases such as HLD or stenosis [1, 2]. Especially epiduroscopic adehisoilysis had good outcome regarding adhesiolysis even if postlumbar surgery syndrome or failed back surgery syndrome had severe adhesion [14–16]. Although PEN with or without epiduroscopy can ameliorate aberrant adhesion and deliver the drugs to target areas such as the nerve root and lumbar disc, it is not possible to decompress a herniated disc with this procedure. Therefore, a new procedure that combines decompression with PEN through the use of a laser system (SELD) has been developed for use in treating spinal lumbar diseases [10, 11, 13, 17]. The main difference between PEN and SELD is, therefore, the ability to perform laser ablation of the disc or adhesion tissue. Accordingly, we compared the efficacy, safety, and change in protruded disc volume with SELD for the treatment of HLD in patients with radicular pain.

Although VAS, ODI, and SF-12 scores improved significantly after each procedure at postoperative week 24 (all *P* < 0.05), improvements of VAS, ODI, and SF-12 and the success rate of Macnab criteria in the SELD group were better than those in the PEN group (all *P* < 0.05). As described in previous studies, SELD enables vaporization of the protruded part of the disc, cauterization of the sinuvertebral nerve, lysis of adhesions near the nerve root, and irrigation of inflamed areas [10]. Consequently, in our study, a comparison between pre- and postoperative MRI images showed that the protruded disc volume decreased significantly by week 24 postoperatively (*P*=0.034). Furthermore, we could confirm the decompression of the nerve root and thecal sac during the SELD procedure.

Laser ablation also enables a contained herniated disc to be physically changed to an uncontained herniated disc. This allows mononuclear cells entering along the margins of the extruded disc to express inflammatory mediators and induce angiogenesis, causing persistent inflammation [18]. An uncontained herniated disc has a possibility of absorption due to dehydration and the inflammation-mediated resorption response because the water content of the disc is higher [19]. Komori et al. [20] noted that the more the herniated nucleus pulposus migrated, the greater the subsequent decrease in size. Henmi et al. [21] reported that large protruded disc fragments diminished more than small fragments. The authors postulated that it may be due to the larger disc fragments having more water content [20, 21]. Based on the reasons discussed above, the results from this study demonstrate that SELD is the superior option for treating HLD in patients with radicular pain compared to PEN.

It is important to note that, in our study, SELD was not always superior to PEN during every follow-up period. At postoperative weeks 4 and 12, for example, the VAS and ODI scores showed no significant differences between the SELD and PEN groups. Rather, the VAS and ODI scores in the SELD group at postoperative week 4 appeared to be higher than those in the PEN group, although not significantly (*P* > 0.05). A previous study reported that because any tissue exposed to the laser is injured microscopically, there were many necrotic cells in all of the tissues where the laser was applied in a parallel as well as perpendicular direction [22]. This finding demonstrated that laser ablation to the disc and adhesion tissue induced an inflammatory reaction. Therefore, it is inferred that, in our study, pain sustained until postoperative week 4 was likely due to this inflammatory reaction [23]. However, there was no difference in SF-12 scores and the success rate of Macnab criteria between the two groups at postoperative week 4.

SELD also has certain disadvantages. A longer operation time is necessary to perform SELD compared with PEN. In addition, because of the possibility of tissue damage, such as thermal injury to the nerve root and dura, it is technically difficult to ablate the protruded disc and perineural adhesions [10, 11, 22]. In this study, there were no complications during the SELD or PEN procedures.

There are some limitations to this study. First, although we performed a comprehensive prospective case-control study, it is nonetheless limited compared to a randomized controlled trial. However, this did not affect the outcome of the SELD group in any way. Second, more studies with larger sample sizes and longer follow-up periods are required to address the relatively small sample size and short-term problems encountered in this study.

## 5. Conclusion

To the best of our knowledge, this is the first comparative study to investigate the differences in outcomes of patients undergoing SELD and PEN for the treatment of HLD. All clinical outcomes were improved in both groups. However, SELD provided a significantly greater clinical success rate than PEN. Furthermore, the volume of protruded lumbar disc decreased significantly after SELD. We suggest that SELD can be used to achieve better outcomes in patients with HLD compared to procedures such as PEN that does not include laser ablation.

## Figures and Tables

**Table 1:** Demographic details of patients segregated into 2 groups based on the treatment procedure.

	SELD (*n* = 28)	PEN (*n* = 40)	*P* value
Age (mean ± SD)	44.8 ± 15.6	50.7 ± 14.0	0.083
Sex (M : F)	15 : 13	15 : 25	0.189
Symptom duration (months, mean ± SD)	6.29 ± 4.95	7.12 ± 4.07	0.378
Level (*n*, %)			0.067
L3/4	1 (3.6)	2 (5)	
L4/5	14 (50)	30 (75)	
L5/S1	13 (36.4)	8 (20)	

SELD = trans-sacral epiduroscopic laser decompression; PEN = percutaneous epidural neuroplasty; L = lumbar; S = sacral.

**Table 2:** Success rate between SELD and PEN groups using Macnab criteria.

Success rate of Macnab criteria (*n*, %)	SELD (*n*=28)	PEN (*n*=40)	*P* value
POD 4 weeks	24 (85.7%)	25 (62.5%)	0.054
POD 12 weeks	27 (96.4%)	18 (45.0%)	≤0.001
POD 24 weeks	27 (96.4%)	20 (50.0%)	≤0.001

Success = excellent and good in Macnab criteria, SELD = trans-sacral epiduroscopic laser decompression; PEN = percutaneous epidural neuroplasty; POD = postoperative duration.

## Data Availability

The data used to support the findings of this study are included in the article.

## Supplementary Materials

Supplementary Materials The protruded disc is sinking during the laser ablation.
